# Bioprinting functional fat – adipocytes as the next frontier in tissue engineering, regenerative medicine, and cultivated meat

**DOI:** 10.3389/fbioe.2026.1923018

**Published:** 2026-09-07

**Authors:** Sophia Nowakowski, Anna Leikeim, Paul Gatenholm, Petra J. Kluger

**Affiliations:** 1 Institute of Interfacial Process Engineering and Plasma Technology (IGVP), Stuttgart, Germany; 2 Faculty of Natural Science, University of Hohenheim, Stuttgart, Germany; 3 Reutlingen Research Institute, Reutlingen University, Reutlingen, Germany; 4 Fraunhofer Institute for Interfacial Engineering and Biotechnology (IGB), Stuttgart, Germany; 5 CELLHEAL AS, Sandvika, Norway; 6 Faculty of Life Sciences, Reutlingen University, Reutlingen, Germany

**Keywords:** adipose tissue bioprinting, biofabrication, cultivated meat, mature adipocytes, microfat

## Abstract

Adipose tissue is a vital component in both regenerative medicine and cultivated meat, yet the selection of the cellular starting material remains an underexplored determinant of adipose construct performance in bioprinting. This mini-review explores next-generation adipose tissue engineering, emphasizing bioprinting functional fat with adipocytes at the intersection of these two fields. While current adipose bioprinting strategies predominantly rely on adipose-derived stem or stromal cells for their expandability and manufacturability, mature adipocytes (ACs) and microfat emerge as alternative cell sources with distinct advantages in functional fidelity, metabolic relevance, and immediate tissue-like behavior. However, these approaches impose fundamentally different biological and engineering constraints that directly influence biofabrication strategy, biomaterial design, and translational potential. This mini review offers a structured comparison of mature adipocyte-, progenitor-, and microtissue-based strategies, highlighting features that influence biofabrication and applicability. Scalability is identified as a major bottleneck in translation, with a focus on process robustness, batch-to-batch reproducibility, and manufacturing concepts relevant to preclinical test systems, clinical applications, and large-scale cultivated meat (CM) production. By proposing a cell-source-guided framework for adipose bioprinting, this mini review aims to provide a practical decision-making perspective for developing next-generation adipose constructs.

## 1 Introduction

Adipose tissue is no longer viewed merely as a passive energy storage organ but as a highly active endocrine and metabolic tissue that plays a central role in tissue homeostasis, regeneration, and systemic signaling. It comprises various cell types, including the most abundant mature adipocytes (ACs), stem cells (adipose-derived stem cells, ASCs), immune cells, and endothelial cells (Duerre and Galmozzi, 2022). Beyond its physiological relevance, adipose tissue has become an increasingly attractive target in bioengineering because of its broad translational potential. In regenerative medicine (RM), engineered adipose tissue is sought for soft-tissue reconstruction and wound repair (Trotzier et al., 2023; El-Mallah et al., 2025), while long-term volume retention and integration remain major clinical challenges. In parallel, adipose cell models are gaining importance for *in vitro* metabolic disease research and drug screening (Contessi Negrini et al., 2024). More recently, engineered fat has also emerged as a critical component of cultivated meat, where adipose tissue contributes to texture, flavor, and nutritional properties (Sugii et al., 2023; Lee et al., 2026).

Despite growing interest, engineering functional adipose tissue remains challenging. Native adipose tissue exhibits delicate architecture, with large, lipid-laden cells embedded in a soft extracellular matrix (ECM) and a dense vascular network that sustains metabolic activity (Soták et al., 2022). These intrinsic biological features impose significant engineering constraints, including buoyancy-related handling challenges, low native mechanical stability, high sensitivity to shear stress, and strong dependence on nutrient and oxygen supply (Blade et al., 2024; Toda et al., 2009). 3D bioprinting offers unprecedented opportunities for the spatially controlled fabrication of adipose constructs, and the field has largely focused on biomaterial formulations and printer modalities (Elango and Zamora-Ledezma, 2025; Zoghi, 2024).

**FIGURE 1:**
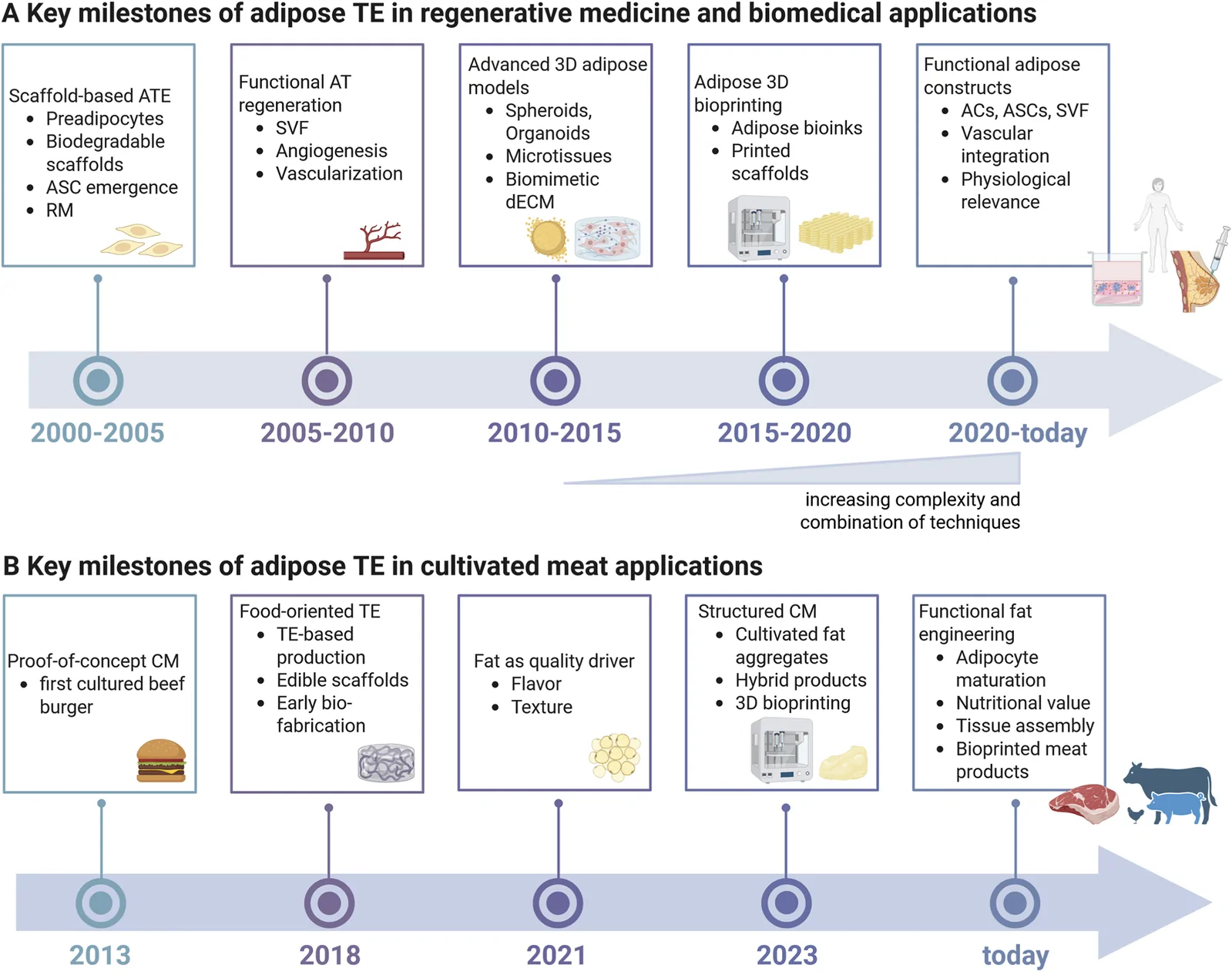
Key milestones of adipose tissue engineering in **(A)** biomedical and **(B)** cultivated meat applications. ATE: adipose tissue engineering, CM: cultivated meat, SVF: stromal vascular fraction, dECM: decellularized extracellular matrix, ACs: mature adipocytes, ASCs: adipose-derived stem cells, RM: regenerative medicine. Created in BioRender. Nowakowski, S. (2026).

However, adipose tissue bioprinting may be less a bioink optimization problem than a challenge posed by cell-biology constraints. ACs and adipogenic differentiated ASCs are often treated as interchangeable building blocks, despite imposing fundamentally different requirements for biofabrication, maturation, and translational implementation. In this mini review, we propose the primary cell source as the critical design criterion for adipose tissue bioprinting. This presents an application-oriented framework that links biological starting material and end-use requirements to guide next-generation adipose tissue engineering in the biomedical and cultivated-meat fields. The development of adipose tissue engineering has been marked by several landmark discoveries and technological innovations, including advances in fat grafting, ASC and SVF-based research, bioprinting and cultivated fat production. Together, these milestones illustrate how regenerative medicine and cultivated meat have converged around the shared goal of generating functional adipose tissue, with Figure 1 summarizing key developments in biomedical applications (Figure 1A) and cultivated meat (Figure 1B).

## 2 Cell sources for biofabrication of adipose tissue–comparison of ASCs, SVF, ACs, and microtissues

Adipose-derived stem cells (ASCs) are among the most widely used cell sources in adipose tissue engineering because of their proliferative capacity, multipotency, and relative ease of isolation. Moreover, ASCs exhibit pronounced paracrine activity, contributing to tissue regeneration beyond their differentiation into mature adipocytes (Gibler et al., 2021; Wang et al., 2025). However, achieving functional maturation requires controlled adipogenic differentiation, which may limit the immediate applicability of this approach in bioprinted constructs (Abbott et al., 2016). ASCs can serve as a versatile tool in adipose tissue bioprinting by providing formats such as single-cell suspensions and compact, high-density cell aggregates (e.g., spheroids), thereby enabling higher cell densities in bioprinted constructs. The use of spheroids also offers potential for upscaling and high-throughput approaches (Colle et al., 2020; Louis et al., 2022).

In addition to isolated ASCs, the stromal vascular fraction (SVF) has emerged as a promising cell source for adipose tissue bioprinting. SVF comprises a heterogeneous mixture of ASCs, endothelial cells, pericytes, immune cells, and other stromal populations, thereby preserving native cell-cell interactions and pro-regenerative signaling. Compared with expanded ASCs, SVF offers enhanced angiogenic and immunomodulatory potential without requiring extensive *in vitro* culture, making it attractive for clinically translatable adipose tissue engineering strategies (Goncharov et al., 2024; Liu et al., 2025). However, its heterogeneous composition can introduce donor-dependent variability and pose challenges for standardization.

Mature adipocytes, by contrast, inherently exhibit the functional characteristics of native adipose tissue, as they are highly differentiated and have immediate endocrine activity (Emont et al., 2022). This makes them particularly attractive for applications requiring immediate functionality. Other characteristics of ACs include their large size (50–150 µm) (Cabeza de Baca et al., 2024), unilocular lipid droplet, and fragile membrane structure, which determine processing conditions. Louis et al. were among the first to show that mature adipocytes are suitable for extrusion-based bioprinting (Louis et al., 2021). Direct comparative studies of ASCs and ACs for bioprinting are scarce. A study by Albrecht et al. demonstrated that both ASCs and ACs can be successfully incorporated into bioprinted constructs, although their behavior and requirements differ significantly (Albrecht et al., 2022). While ASCs exhibit greater robustness and adaptability during processing, mature adipocytes offer superior immediate functionality and physiological relevance. However, the study did not examine long-term functionality or a specific application. This highlights a fundamental trade-off between printability and functionality, a key consideration in the design of adipose tissue constructs.

In clinical practice, adipose tissue can also be incorporated into engineering workflows as higher-order tissue formats, such as microfat, which consists of mechanically processed adipose tissue fragments that preserve native multicellular complexity. Microfat retains ACs alongside progenitor cells, stromal and vascular-associated cell populations, and ECM components (Parsons et al., 2025). As an autologous tissue source, microfat is attractive for soft-tissue reconstruction because it combines biological complexity with clinical accessibility. Autologous adipose tissue can be obtained by lipoaspiration, a clinically established and comparatively straightforward procedure. Since the refinement of lipoaspirate processing by Coleman and Saboeiro (Coleman and Saboeiro, 2007), it has been widely used in aesthetic surgery as fat transfer, in which mature adipocytes, preadipocytes, and stromal vascular fractions are injected. Injection of autologous fat does not cause a foreign body reaction, but the injected adipose tissue is resorbed by up to 75% over a 6-month period (Gause et al., 2014). This resorption is primarily attributed to adipose cell necrosis within the injected volume due to insufficient diffusion of nutrients and oxygen. Mechanical fragmentation of lipoaspirate into microfat has therefore been developed to improve fat transfer outcomes. Several commercially available processing devices are used to generate microfragmented adipose tissue for clinical applications (Bianchi et al., 2013; Schmitt et al., 2021; Svolacchia and Svolacchia, 2026).

## 3 Shared engineering constraints in adipose tissue bioprinting–cell source as a design determinant

Adipose tissue bioprinting is constrained by engineering challenges that are fundamentally shaped by the biological properties of the selected cell source. While isolated ASCs and ACs can both serve as adipogenic building blocks, intermediate formats such as microfat have emerged as biologically attractive alternatives (Jeong et al., 2025). These formats, with their distinct cellular characteristics, impose markedly different requirements for process design, construct maturation, and translational feasibility.

A primary challenge lies in printability and process robustness, particularly regarding mechanical handling and shear-stress tolerance. Extrusion-based bioprinting subjects biological components to pressure gradients, nozzle-induced deformation, and transient mechanical stress, all of which can compromise viability and function (Chand et al., 2022). Isolated ACs, due to their large size and fragile structure, are especially vulnerable to shear-induced rupture, which substantially narrows the printable rheological window and lowers resolution (Albrecht et al., 2022). In contrast, ASCs and SVF demonstrate greater mechanical resilience and compatibility with conventional bioprinting workflows. Microtissue-based approaches occupy an intermediate position: the biological complexity they entail creates significant engineering challenges. While microfat preserves native cell-cell and cell-matrix interactions that may enhance functionality, its heterogeneous size distribution, mechanical fragility, and therefore increased risk of nozzle clogging create additional handling and reproducibility challenges (Schmitt et al., 2021; Lee et al., 2025). However, microfat itself lacks the mechanical properties that are required for reconstructing soft tissues and still undergoes volume resorption.

These cell-source-specific constraints directly extend to bioink design. In adipose tissue bioprinting, bioinks can broadly be grouped into natural biomaterial-based (e.g., collagen, gelatin, hyaluronic acid, fibrin, alginate), synthetic polymer-based (e.g., polyethylene glycol derivatives), and composite/hybrid bioinks that combine biological activity with tunable mechanical properties (Chandarana et al., 2026). Recent advances have introduced tissue-specific decellularized extracellular matrix (dECM) bioinks that incorporate adipogenic factors to better recapitulate the microenvironment of native adipose tissue (Lee et al., 2021; Kim et al., 2021; Cui et al., 2025). Across these categories, soft hydrogel formulations remain particularly relevant due to their ability to preserve adipocyte viability and metabolic function while supporting long-term tissue remodeling. Accordingly, bioinks for mature adipocytes and adipose microtissues must prioritize cell protection, low crosslinking stress, and long-term preservation of metabolic phenotype within soft matrices. The buoyancy of ACs makes bioink formulations prone to phase separation, requiring careful optimization of mixing protocols (Albrecht et al., 2022). Beyond that, ASC- and scaffold-based formulations must provide instructive biochemical and mechanical cues to support adipogenic differentiation and tissue maturation (Zhang et al., 2018). Bioink design for microfat must balance structural support with minimal mechanical disruption, while accommodating larger, less standardized biological components (Schmitt et al., 2021; Jeong et al., 2025).

All biofabrication strategies remain constrained by nutrient supply and mass transport limitations, as native adipose tissue is highly vascularized and metabolically demanding. This challenge becomes particularly acute in large AC- or microtissue-rich constructs, where diffusion limitations rapidly impair viability (Louis et al., 2021).

Collectively, adipose tissue bioprinting reflects a broader fidelity-versus-manufacturability trade-off summarized in Table 1: isolated ACs offer high physiological relevance but poor process robustness; ASCs provide manufacturability and scalability but delayed functionality; SVF represents an intermediate strategy that retains multicellular complexity and pro-regenerative signaling while avoiding extensive cell expansion; and microfat-based approaches may offer a translationally attractive middle ground by partially preserving native tissue complexity while remaining technically manageable.

**TABLE 1:** Selection criteria of different cell sources for adipose tissue bioprinting.

Criterion	Adipose-derived stem cells	Stromal vascular fraction	Mature adipocytes	Microfat/microtissues
Availability/expansion	High (expandable *in vitro*)	Moderate (limited expansion as a mixed population)	Limited (non-proliferative)	Moderate (donor-dependent)
Initial functionality	Low (requires differentiation)	Medium (contains adipogenic, endothelial and stromal components)	High (immediate lipid function)	Very high (native tissue function)
Time-to-function	High (differentiation for weeks)	Low-medium (functionality present immediately, maturation may improve performance)	Low (ready-to-use after isolation)	Low (ready-to-use after isolation)
Phenotype fidelity	Medium (depends on protocol)	High (native cellular heterogeneity)	High (native adipose phenotype)	Very high (multi-cellular, ECM included)
Shear robustness	High	Medium	Low (fragile)	Medium (context-dependent)
Processability/bioprinting	High (adaptable)	Medium-high (good handling)	Challenging (buoyancy, shear sensitivity)	Low-medium (bulk handling)
Long-term stability	Medium (risk of drift)	Medium-high	Low-medium (risk of dedifferentiation)	High (clinically proven persistence)
Batch variability	Medium-high	High (donor + processing variability)	High	High (donor + processing variability)
Scalability	High	Medium	Low	Low
System complexity	Low (single-cell focus)	Medium-high (multi-cell-population)	Low-medium	High (multi-component system)
Regulatory complexity	Medium	High (heterogeneous cell population)	Medium-high	High (heterogeneous composition)
Application relevance	Early-stage biofabrication (TE) Industrial CM production	Vascularized adipose tissue engineering Translational RM	High-fidelity adipose function models (TE, RM) High-end CM (in combination with ASCs)	Clinically inspired constructs Translational RM

## 4 Design considerations for bioprinted adipose tissue by application in tissue engineering, regenerative medicine, and cultivated meat

In tissue engineering applications, particularly for *in vitro* metabolic and disease models, the primary objective is to recreate physiologically relevant adipose tissue function rather than to replace tissue. Consequently, bioprinted adipose tissue constructs must exhibit authentic endocrine behavior, metabolic responsiveness, and long-term phenotype stability. Key functional requirements include adipokine secretion, insulin sensitivity, lipolytic activity, and appropriate responses to inflammatory and metabolic stimuli. In this context, cell source selection is particularly critical, as disease-associated phenotypes may be intrinsically encoded within donor-derived adipose tissue (Hinte et al., 2024). ACs provide immediate functionality and may preserve disease-associated transcriptional and metabolic signatures acquired *in vivo*, thereby enabling patient-relevant modeling of adipose dysfunction. Recent studies have demonstrated that AC-based 3D adipose models recapitulate key hallmarks of obesity-associated dysfunction, including altered lipid metabolism, hypertrophy, and inflammatory responses (Ahn et al., 2022; Rogal et al., 2022). In contrast, ASC-based constructs require previous cell expansion and differentiation, which improves experimental reproducibility and scalability but may attenuate donor-specific characteristics and delay functional maturity.

The requirements for vascularized adipose tissue models shift substantially, as physiological crosstalk between adipocytes and the microvasculature becomes essential. In these models, establishing stable, perfusable networks and maintaining long-term nutrient and oxygen supply are often more important than maximizing throughput. Vascularized bioprinted adipose tissue models containing ACs, endothelial cells, and supporting stromal cells have demonstrated preserved fatty acid uptake and adipose-specific functionality, underscoring the importance of recreating the native adipose niche (Louis et al., 2021; Goldfracht et al., 2024).

By contrast, high-throughput drug screening platforms prioritize standardization, reproducibility, scalability, and compatibility with automated workflows. In these systems, assay robustness, batch-to-batch consistency, and cost efficiency often outweigh the need to fully reproduce patient-specific adipose biology. Here, bioprinting enables the generation of standardized adipose tissue models and miniaturized tissue arrays suitable for parallelized testing. ASC-derived models are therefore attractive for their scalability and manufacturing consistency, whereas AC-based screening systems are primarily advantageous when compound responses depend on preserving donor-specific metabolic phenotypes. Notably, high-throughput adipose screening platforms based on patient-derived ACs have already demonstrated the feasibility of combining physiological relevance with scalable drug testing, illustrating the potential of AC-containing models for precision medicine applications (Louis et al., 2021).

In regenerative medicine, bioprinted adipose constructs composed of microtissues hold great potential for soft-tissue reconstruction, wound healing, and the treatment of volumetric defects. The team at Sahlgrenska University Hospital and Chalmers in Sweden showed that human microfragmented adipose tissue (microfat) can be combined with tunicate nanocellulose/alginate hydrogels to generate viable, biocompatible, dimensionally stable, and vascularized adipose tissue grafts *in vivo* (Säljö et al., 2022; Säljö et al., 2020; Oskarsdotter et al., 2023). In these constructs, the hydrogel separates adipose tissue fragments while allowing diffusion of oxygen and nutrients, and the retained microvascular components contribute to vascularization after implantation (Apelgren et al., 2022). These findings support the translational potential of microfat-based bioprinting for soft-tissue reconstruction; however, clinical implementation remains constrained by regulatory and manufacturing requirements. Clinical translation of adipose tissue bioprinting is challenged by regulatory and manufacturing requirements. While most cell-based bioprinting approaches involving cell expansion or differentiation are classified as Advanced Therapy Medicinal Products (ATMPs), minimally manipulated adipose tissue products, including microfat, nanofat, and SVF gel, may facilitate clinical translation when used in homologous applications (Bonini et al., 2026; Jiao et al., 2026). Microfat consists of mechanically processed adipose tissue fragments that largely retain ACs and ECM. In contrast, nanofat and SVF gel are generated through more extensive mechanical processing, resulting in the depletion of ACs and the enrichment of stromal, progenitor, and vascular-associated cell populations, making them primarily regenerative rather than volumizing products (Arcani et al., 2026). Scaling to clinically relevant volumes, such as breast reconstruction, nevertheless requires improvements in printing speed, shape fidelity, crosslinking strategies, and vascularization to ensure long-term graft survival (Jeong et al., 2025).

The cultivated meat field has progressed from early proof-of-concept studies to pilot-scale processes and first regulatory approvals in selected markets; however, broad commercial implementation remains limited by production costs, the development of serum-free and food-grade media, scalable bioprocessing, regulatory pathways, and consumer acceptance (Khan et al., 2025). Within this field, adipose tissue engineering has gained increasing attention because fat is a major determinant of flavor, juiciness, mouthfeel, and nutritional profile, yet scalable production, maturation, and standardization of cultivated fat remain less established than muscle-focused approaches (Sugii et al., 2023; Lee et al., 2026). Accordingly, current cultivated-fat strategies are increasingly centered on expandable progenitor cells and controlled adipogenic differentiation, while mature adipocytes and adipose microtissues remain valuable benchmarks for biological functionality rather than scalable production inputs (Sugii et al., 2023; Lee et al., 2026; Yuen et al., 2022). Bioprinting is being explored for spatial organization and hybrid meat structuring; however, conventional nozzle-based approaches may be difficult to scale industrially. High-throughput alternatives such as 3D bio-screen printing have recently been proposed for edible, meat-like scaffolds and multilayered hybrid structures, although their applicability to adipose tissue constructs remains to be demonstrated (Maatz et al., 2026).

Against this background, cultivated meat imposes fundamentally different requirements on adipose tissue engineering. The primary objective is not tissue integration, but the scalable production of fat that contributes to sensory and nutritional quality. In this context, “functional fat” is defined by parameters such as lipid composition, lipid accumulation, and reproducibility, all of which directly influence flavor and texture (Sugii et al., 2023; Lee et al., 2026). Long-term physiological integration and host interaction, critical in biomedical applications, are of secondary importance. This highlights a fundamental paradigm shift: while regenerative medicine aims to recreate living systems, cultivated meat requires more robust, scalable production of functional components under industrial constraints.

Progenitor-based strategies, particularly those using ASCs, represent the most viable approach, as their proliferative capacity enables the large-scale cell production required for industrial manufacturing (Yuen et al., 2022; Louis et al., 2023). However, functional maturation via adipogenic differentiation remains a bottleneck, as it is time-consuming and often yields heterogeneous outcomes that can affect lipid composition and overall product consistency (Sugii et al., 2023; Lee et al., 2026). Importantly, these constraints also directly affect bioprinting strategies. In the context of biofabrication, scalable cell expansion and process robustness are key determinants of printability, cell-loading density, and the reproducibility of printed constructs.

In contrast, the immediate functionality of mature adipocytes is outweighed by their lack of proliferative capacity, which inherently limits their scalability, and by challenges in process compatibility. As a result, mature adipocytes require continuous sourcing from primary tissue, which conflicts with the goal of scalable, animal-independent production (Sugii et al., 2023). In addition, their large size, buoyancy, and mechanical fragility complicate handling and integration into robust manufacturing processes (Dufau et al., 2021; Lauschke and Hagberg, 2023). As such, mature adipocytes represent a biologically relevant but industrially poorly scalable cell source for cultivated fat production. This further limits their compatibility with bioprinting workflows, which rely on reproducible, high-throughput processing conditions. A similar limitation applies to microfat. While it represents a physiologically relevant and clinically established adipose tissue format, its direct use in cultivated meat is not feasible due to inherent limitations in scalability, standardization, and sourcing (Yuen et al., 2022). Therefore, microfat is better viewed as a conceptual reference for tissue complexity than a practical production strategy.

Materials and processing impose additional constraints: All components must be edible, scalable, and cost-effective, which limits the applicability of many biomaterials commonly used in biomedical contexts (Moslemy et al., 2023; Seibert et al., 2025). Additionally, production processes must be compatible with large-scale bioreactor systems and highly reproducible, placing a strong emphasis on process robustness and manufacturability (Yuen et al., 2022). Taken together, scalability requirements fundamentally constrain cell source selection in cultivated meat applications. Although mature adipocytes and microfat offer valuable insights into adipose tissue function, they do not align with the fundamental paradigm of cultivated meat production. Progenitor-based systems currently offer the only realistic route toward scalable manufacturing, though they require optimization to achieve the desired functional properties (Sugii et al., 2023; Yuen et al., 2022). These trade-offs underscore the necessity of strategies that balance scalable cell expansion with enhanced control over adipogenic differentiation and lipid quality.

## 5 Key unresolved challenges and future directions

The challenges outlined above highlight the need for a more integrated and standardized framework across studies and applications. A major issue remains the lack of standardized phenotyping panels for ASCs and ACs, as even in controlled studies ASCs are defined using heterogeneous marker sets and adipogenic outcomes (Khazaei et al., 2022), complicating reproducibility across bioprinting workflows where process parameters add another layer of variability. This is particularly problematic, given that bioprinting-induced shear stress and bioink composition can directly influence phenotype, reinforcing the need for harmonized quality-control metrics to improve cross-study comparability (Zhao et al., 2026).

In parallel, the long-term stability and maintenance of phenotype in engineered adipose tissue remain unresolved. Mature adipocytes provide immediate functionality but exhibit limited stability *in vitro*, whereas progenitor-based systems require robust and reproducible differentiation to achieve consistent functional outcomes (Berger et al., 2021). Reliable metrics capturing long-term functionality, including lipid metabolism and phenotype persistence, are still lacking.

Material design represents an additional bottleneck that is inherently application-dependent. In regenerative medicine, biomaterials must be biocompatible, resorbable, and compliant with clinical requirements, whereas cultivated meat demands edible, scalable, and cost-effective materials. This divergence fundamentally limits the transferability of existing biofabrication strategies (Moslemy et al., 2023). Scalability and reproducibility of cell sourcing remain critical constraints. Progenitor-based systems enable expansion but suffer from variability in differentiation outcomes, while mature adipocytes and microtissues offer high functional fidelity but are restricted by sourcing limitations and process integration. In particular, achieving consistent adipogenic differentiation and tissue assembly at scale remains a major unresolved challenge (Sugii et al., 2023; Yuen et al., 2022).

Finally, a shift toward system-level design approaches is required. Current strategies often focus on optimizing individual components, such as cell type or biomaterial. However, native adipose tissue functions as a multicellular, structurally organized system. Future biofabrication strategies may therefore benefit from incorporating microfat-inspired design principles, including cellular heterogeneity and matrix integration, while maintaining compatibility with application-specific process constraints. Addressing these interconnected challenges will require integrated approaches that combine cell biology, materials science, and bioprocess engineering to enable robust, scalable, and application-specific adipose tissue constructs.

## 6 Conclusion

Despite significant advances, one cell source cannot meet all requirements for functional adipose tissue bioprinting. Future strategies may therefore focus on combining different cell types or integrating co-culture systems to balance structural stability with physiological functionality. Mature adipocytes, ASCs, SVF, and microfat represent a continuum rather than isolated options, with microfat as a critical translational anchor, especially for clinical applications. This is contrasted in the different fields of regenerative medicine and cultivated meat: while regenerative applications aim to recreate living systems by integrating tissues, cultivated meat requires robust, scalable production of functional components under industrial constraints. This application-dependent perspective highlights that the choice of cell source will depend on the specific application, underscoring the need for tailored multi-scale design strategies in adipose tissue bioprinting.
